# Blood Pressure Control in Aging Predicts Cerebral Atrophy Related to Small-Vessel White Matter Lesions

**DOI:** 10.3389/fnagi.2017.00132

**Published:** 2017-05-15

**Authors:** Kyle C. Kern, Clinton B. Wright, Kaitlin L. Bergfield, Megan C. Fitzhugh, Kewei Chen, James R. Moeller, Nooshin Nabizadeh, Mitchell S. V. Elkind, Ralph L. Sacco, Yaakov Stern, Charles S. DeCarli, Gene E. Alexander

**Affiliations:** ^1^Department of Neurology, Evelyn F. McKnight Brain Institute, University of Miami Miller School of MedicineMiami, FL, USA; ^2^Neuroscience and Physiological Sciences Graduate Interdisciplinary Programs, University of ArizonaTucson, AZ, USA; ^3^Department of Psychology and Evelyn F. McKnight Brain Institute, University of ArizonaTucson, AZ, USA; ^4^Computational Image Analysis Program, Banner Alzheimer InstitutePhoenix, AZ, USA; ^5^School of Mathematics and Statistics, Arizona State UniversityTempe, AZ, USA; ^6^Arizona Alzheimers ConsortiumPhoenix, AZ, USA; ^7^Department of Psychiatry, College of Physicians and Surgeons, Columbia UniversityNew York, NY, USA; ^8^Department of Neurology, College of Physicians and Surgeons, Columbia UniversityNew York, NY, USA; ^9^Department of Neurology and Center for Neuroscience, University of California, DavisDavis, CA, USA; ^10^Department of Psychiatry and BIO5 Institute, University of ArizonaTucson, AZ, USA

**Keywords:** white matter hyperintensities, brain atrophy, hypertension, cerebrovascular disease, cognition, aging, scaled subprofile model, voxel-based morphometry

## Abstract

Cerebral small-vessel damage manifests as white matter hyperintensities and cerebral atrophy on brain MRI and is associated with aging, cognitive decline and dementia. We sought to examine the interrelationship of these imaging biomarkers and the influence of hypertension in older individuals. We used a multivariate spatial covariance neuroimaging technique to localize the effects of white matter lesion load on regional gray matter volume and assessed the role of blood pressure control, age and education on this relationship. Using a case-control design matching for age, gender, and educational attainment we selected 64 participants with normal blood pressure, controlled hypertension or uncontrolled hypertension from the Northern Manhattan Study cohort. We applied gray matter voxel-based morphometry with the scaled subprofile model to (1) identify regional covariance patterns of gray matter volume differences associated with white matter lesion load, (2) compare this relationship across blood pressure groups, and (3) relate it to cognitive performance. In this group of participants aged 60–86 years, we identified a pattern of reduced gray matter volume associated with white matter lesion load in bilateral temporal-parietal regions with relative preservation of volume in the basal forebrain, thalami and cingulate cortex. This pattern was expressed most in the uncontrolled hypertension group and least in the normotensives, but was also more evident in older and more educated individuals. Expression of this pattern was associated with worse performance in executive function and memory. In summary, white matter lesions from small-vessel disease are associated with a regional pattern of gray matter atrophy that is mitigated by blood pressure control, exacerbated by aging, and associated with cognitive performance.

## Introduction

White matter hyperintensities (WMH) observed on T2 MRI are frequently discovered incidentally but are more prevalent with increased age, hypertension, and other cerebrovascular risk factors (Fazekas, 1989; DeCarli et al., 1995; de Leeuw et al., 2001). Although, the origins of WMHs are thought to be heterogeneous, ischemic small-vessel disease is one established etiology (DeCarli et al., 2005), and extensive lesions are associated with an increased risk of stroke (Fazekas et al., 1993), gait disturbance (Whitman et al., 2001), cognitive impairment and decline (de Groot et al., 2000), and dementia (Barber et al., 1999).

Hypertension (Salerno et al., 1992), subclinical elevated blood pressure (DeCarli et al., 1999), and WMH (Wen et al., 2006) are also associated with reduced gray matter volume (GMV). WMH and hypertension increase the risk of all-cause dementia (Barber et al., 1999; Launer et al., 2000), Alzheimer's disease (Hofman et al., 1997), and are independently linked to cognitive decline (van Swieten et al., 1991), with aspects of frontal lobe-mediated executive functions, memory, and processing speed preferentially affected (Junqué et al., 1990; Prins et al., 2005). Volumetric studies have found frontal and temporal lobe atrophy associated with WMH and aging (Raz et al., 2003; Raji et al., 2012), but the complex relationship between WMH, gray matter atrophy, cerebrovascular risk factors, and cognition is poorly understood. Since cognitive changes in aging may be mediated by gray matter loss (Raji et al., 2012), it is important to understand how WMH relate to gray matter volume, and which risk factors modify this relationship.

In this study we identify regional differences in GMV associated with WMH lesion load in a group of cognitively normal older adults and investigate the effects of blood pressure control, age, sex, education, and overall intelligence on this relationship, as well as examine the association between the WMH-associated GMV (WMH~GMV) pattern and domain-specific cognitive performance.

## Methods

### Participants

The Northern Manhattan Study (NOMAS) was designed to determine stroke incidence, risk factors, and prognosis in a race-ethnically diverse urban population. Study details have been published previously. (Sacco et al., 1997) Briefly, eligible participants were: (a) stroke-free; (b) greater than 40 years of age; and (c) residents of Northern Manhattan for at least 3 months in a household with a telephone and were enrolled between 1993 and 2001 (*N* = 3,298). Participants older than 50 years who remained stroke-free were invited to participate in a brain MRI substudy during annual telephone follow-up between 2003 and 2008. In addition, 199 household members of participants that met all NOMAS inclusion/exclusion criteria were enrolled to reach a total sample of 1,290. The study was approved by the Institutional Review Boards of Columbia University and the University of Miami and the protocol was designed and carried out in accordance with their recommendations. All participants provided informed written consent.

### Sample selection

For the current study, we identified 64 participants with normotension, controlled hypertension or uncontrolled hypertension. We obtained systolic and diastolic blood pressures measured at the brachial artery while seated with a mercury sphygmomanometer after a period of rest for 10 min. Two measurements were taken at least 1 h apart, and the values were averaged. Participants with a reported history of hypertension on antihypertensive medication were classified as having controlled hypertension if the mean blood pressure was less than 140 systolic and 90 diastolic. Participants were classified as uncontrolled hypertension if they had a previous diagnosis of hypertension and the mean blood pressure was greater than 140 systolic or 90 diastolic. Normotensive patients had no history of hypertension and were not taking antihypertensive medications. Groups were case-wise matched for age, sex, race, and educational attainment. Participants were classified as having finished 8th grade or less. The current study participants were similar to and reflective of the overall NOMAS population.

### Cognitive assessment

Participants in the NOMAS MRI substudy were administered a neuropsychological battery in either English or Spanish by trained research assistants on the day of MRI acquisition. As reported previously by Siedlecki et al. (2009) cognitive domains were created using an exploratory factor analysis to group tests with similar variance across a cohort of 796 participants without cognitive impairment. The cognitive domains in this study included memory, executive function, processing speed, language, and general intellectual ability (Gc) (Siedlecki et al., 2009). The neuropsychological tests used to represent each cognitive domain are in keeping with recommendations from the NINDS Canadian Stroke Network Vascular Cognitive Impairment Harmonization Standards. The memory domain was comprised of the components of a modified California Verbal Learning Test. Executive function was assessed with the Wechsler Adult Intelligence Scale letter-number sequencing test, the digit ordering task, the odd-man-out task, and a difference score from the Color Trails test (similar to Trailmaking Test form B). Processing speed was evaluated with the Grooved Pegboard task. Language (not previously reported) was assessed with the Controlled Oral Word Association Task, the Animal Naming test, and the Boston naming test. Finally, to estimate Gc (Cattell, 1987) as a surrogate for premorbid IQ (Siedlecki et al., 2009), we administered the Peabody Picture Vocabulary Test. Subtest scores were converted into Z-scores for each of the four domains based on the mean and standard deviation of all participants of the NOMAS brain MRI substudy. A self-reported depression scale, The Center for Epidemiologic Studies Depression Scale (CESD), was available in 58 of 64 participants. The CESD is a 20-question survey of depressive symptoms that was developed in 1977 and has been used in diverse populations for the epidemiologic study of depression (Radloff, 1977). ApoE genetic testing was available for a subgroup of 58 of the 64 participants from fasting blood samples drawn at the time of MRI. ApoE alleles were determined by *Hha*I digestion of PCR products amplified from genomic DNA as described previously. Carriers of one or two alleles of ApoE-4 were collapsed into the same category and compared against carriers of only ApoE-3 or ApoE-2 (Hixson and Vernier, 1990; Willey et al., 2014).

### Image acquisition and processing

Participants were scanned with a 1.5T scanner (Phillips, The Netherlands). A 3D T1 structural image (slice thickness 1.5 mm no gap, TE 2.1 ms, TR 20 ms, flip angle 20°) was acquired to assess gray matter volume (GMV). A T2 FLAIR sequence (FOV 250 mm, matrix 192 × 133 scaled to 256 × 256, slice thickness 3 mm no gap, TE 144 ms, TR 5,500 ms, TI 1900 ms, flip angle 90°) was used to identify WMH, total brain volumes (TBV), and total intracranial volumes (TIV), which were obtained using a validated, automatic segmentation algorithm as previously reported (DeCarli et al., 1992; Wright et al., 2005). GMV was converted into a fraction of TBV (GMF) for comparison. WMH volumes were converted into a percentage of TIV to correct for head size, and then log transformed (logWMH) to use in regression analyses, since raw volumes were not normally distributed.

T1 images were processed using SPM8 (Wellcome Department of Imaging Neuroscience, London, United Kingdom; www.fil.ion.ucl.ac.uk/spm) Voxel Based Morphometry (VBM) (Ashburner and Friston, 2000) software to segment MRIs into gray matter, white matter and CSF after image intensity nonuniformity correction. The DARTEL (Diffeomorphic Anatomic Registration Through Exponentiated Lie algebra) toolbox was used to create a group-specific brain template aligned to a common MNI (Montreal Neurologic Institute) space by iteratively aligning tissue segmentations using a common tissue probability map. The gray matter maps were processed to preserve volume information with the spatial deformations. A 10 mm smoothing kernel was applied and statistics were performed on gray matter volume (GMV) maps across the sample at each voxel.

We used the Scaled Subprofile Model (SSM) (Moeller et al., 1987; Alexander and Moeller, 1994) to identify the regional pattern of VBM GMV differences associated with WMH load in the cohort. The SSM is a multivariate technique that tests for regional covariance patterns in neuroimaging scans without requiring conservative correction for multiple comparisons on a voxel-wise basis. This analytic method has been applied to numerous functional and structural imaging studies with PET and MRI (Eidelberg et al., 1995; Alexander et al., 1999; Smith et al., 2006). Applications to MRI VBM have demonstrated sensitivity in identifying regional patterns of gray matter changes related to aging and risk for Alzhiemer's disease (Alexander et al., 2006, 2008, 2012; Bergfield et al., 2010).

In our study, we used VBM and SSM to identify a pattern of GMV variability associated with logWMH volume and created a set of SSM subject scores that reflect the extent to which each individual expresses this WMH-related gray matter volume (WMH~GMV) pattern. Individual subject scores were used in subsequent group analyses.

The SSM analysis was performed using MATLAB (Math Works, Natick, Massachusetts, USA). GMV images were smoothed and log transformed, and means across regions and participants were subtracted at each voxel. A Principal Component Analysis was performed to decompose the dataset into gray matter network components that explain the greatest variance in the neuroimaging data. To select the linear combination of gray matter network components that best model the variance in WMH across the cohort, we used the Akaike Information Criterion (AIC) (Burnham and Anderson, 2002) with multiple-regression. The first subset of components that together mapped the lowest AIC value in the regression model to identify the WMH~GMV network was selected as the best set of component predictors. Total intracranial volume (TIV) was included as a covariate to account for differences in head size as previously described (Bergfield et al., 2010). To provide reliability estimates of the resulting WMH~GMV pattern, we used bootstrap resampling with 500 iterations of the SSM multivariate regression, and used the means and standard deviations to calculate a Z-score at each voxel (Alexander et al., 2012). Thresholding at *Z* ≤ −2 and *Z* ≥ 2 created Z-maps identifying areas of GMV differences robustly associated with logWMH.

### Statistics

Group differences were determined using ANOVA and chi-squared tests. Individual subject scores were calculated from the SSM multivariate regression that reflect the extent to which each participant expresses the WMH~GMV pattern. These values were used to compare the strength of the WMH~GMV association between uncontrolled hypertensive, controlled hypertensive, and normotensive participants using ANCOVA and ordinal linear regression while controlling for age and educational level. We tested the effects of age, sex, educational attainment and Gc on this relationship using multivariate linear regression. We also tested the association between expression of the WMH~GMV pattern and cognitive performance using multivariate regression models that included the pattern subject scores and cognitive domain Z-scores for memory, executive function, processing speed, and language while controlling for age and level of education. A conservative bonferroni correction was applied to these 4 multivariate linear regression models for multiple comparisons and the corrected *p*-values reflect this. All tests were two-tailed and an alpha of 0.05 determined significance.

## Results

Participants included 64 adults ranging from 60 to 86 years of age (mean = 72 years). Twenty-two had uncontrolled hypertension, 21 had controlled hypertension, and 21 were normotensive. Groups were matched case-wise for age, sex, race and education. There were no differences between the study participants and the overall NOMAS population in age, ethnic distribution, smoking status, diabetes status, cognitive scores or CESD. There were no group differences in Gc or performance in each of the cognitive domains. Mean cognitive Z-scores were close to zero for each group and the mean of the four cognitive domains was greater than −1.5 for each participant, indicating a level of cognitive function representative of the NOMAS study without significant cognitive impairment. There were no group differences in GMF, WMH volume, or total brain volume (TBV) after adjusting for head size with TIV. (Table 1)

**Table 1:** **Clinical Characteristics**.

	**Total**	**Normotensives**	**Controlled hypertensives**	**Uncontrolled hypertensives**	*P*-values^*^
N	64	21	22	21	
Age in years mean ± SD	72 ± 7	72 ± 7	71 ± 8	71 ± 7	0.80
Males:Female	21:43	7:14	7:15	7:14	0.99
never:former:current smoker	31:26:7	10:10:1	9:9:4	12:7:2	0.57
# with Diabetes	14	3	5	6	0.53
# with PVD	10	1	5	4	0.24
# with <8th grade Education	35	12	12	11	0.95
IQ Z-score mean ± SD	0.08 ± 0.81	0.13 ± 0.97	0.06 ± 0.80	0.06 ± 0.68	0.95
Memory Z-score mean ± SD	0.07 ± 0.79	0.03 ± 0.88	0.15 ± 0.75	0.03 ± 0.73	0.85
Executive Function Z-score mean ± SD	–0.14 ± 0.81	–0.12 ± 0.84	–0.07 ± 0.80	–0.24 ± 0.81	0.80
Processing Speed Z-score mean ± SD	0.01 ± 0.35	–0.03 ± 0.30	0.07 ± 0.40	–0.02 ± 0.34	0.54
Language Z-score mean ± SD	0.01 ± 0.75	0.15 ± 0.89	–0.01 ± 0.69	–0.11 ± 0.67	0.54
Total Cerebral Volume cc ± SD	1138 ± 118	1137 ± 99	1130 ± 118	1146 ± 139	0.91
Gray Matter Fraction ± SD	0.56 ± 0.02	0.57 ± 0.02	0.56 ± 0.02	0.56 ± 0.02	0.75
WMH Volume cc mean ± SD	7.7 ± 9.1	5.6 ± 7.7	9.0 ± 8.3	8.4 ± 11.1	0.45
WMH Brain Fraction mean ± SD %^†^	0.66 ± 0.73 %	0.48 ± 0.61%	0.78 ± 0.70%	0.70 ± 0.85%	0.38

*WMH, white matter hyperintensities; SD, standard deviation; PVD, peripheral vascular disease*.

* *P-values reflect Chi-squared test for gender, smoking, diabetes, PVD, and education, One-way ANOVA for all others*.

† *Total WMH Volume/Total Intracranial Volume*.

Normotensive participants were not on antihypertensive medications. In the controlled hypertension group, all participants were on at least one antihypertensive medication and 5 participants were on 2 antihypertensives. In the uncontrolled hypertensive group, 9 participants were not on antihypertensive medication, 6 were on one antihypertensive, 6 were on 2 antihypertensives, and 1 was on 3 antihypertensives (Supplementary Table). The distribution of antihypertensive medication class was not different between the participants with controlled and uncontrolled hypertension.

More educated participants had better executive function and language scores (*p* < 0.0001) and tended to have non-significantly better memory (*p* = 0.07) and processing speed (*p* = 0.10) than those with less education. Participants with higher education had a trend for greater WMH load (*p* = 0.07) but no differences in adjusted TBV.

**Figure 1:**
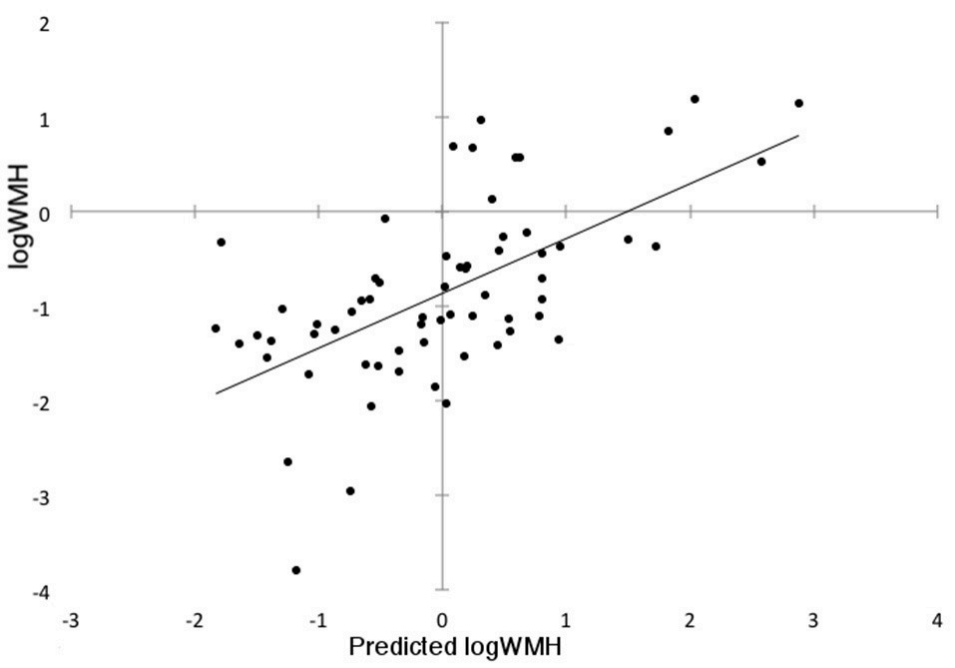
**Subject scores calculated from the Scaled Subprofile Model (SSM) gray matter volume covariance pattern predict log of white matter hyperintensity (WMH) volume across the cohort**.

**Figure 2:**
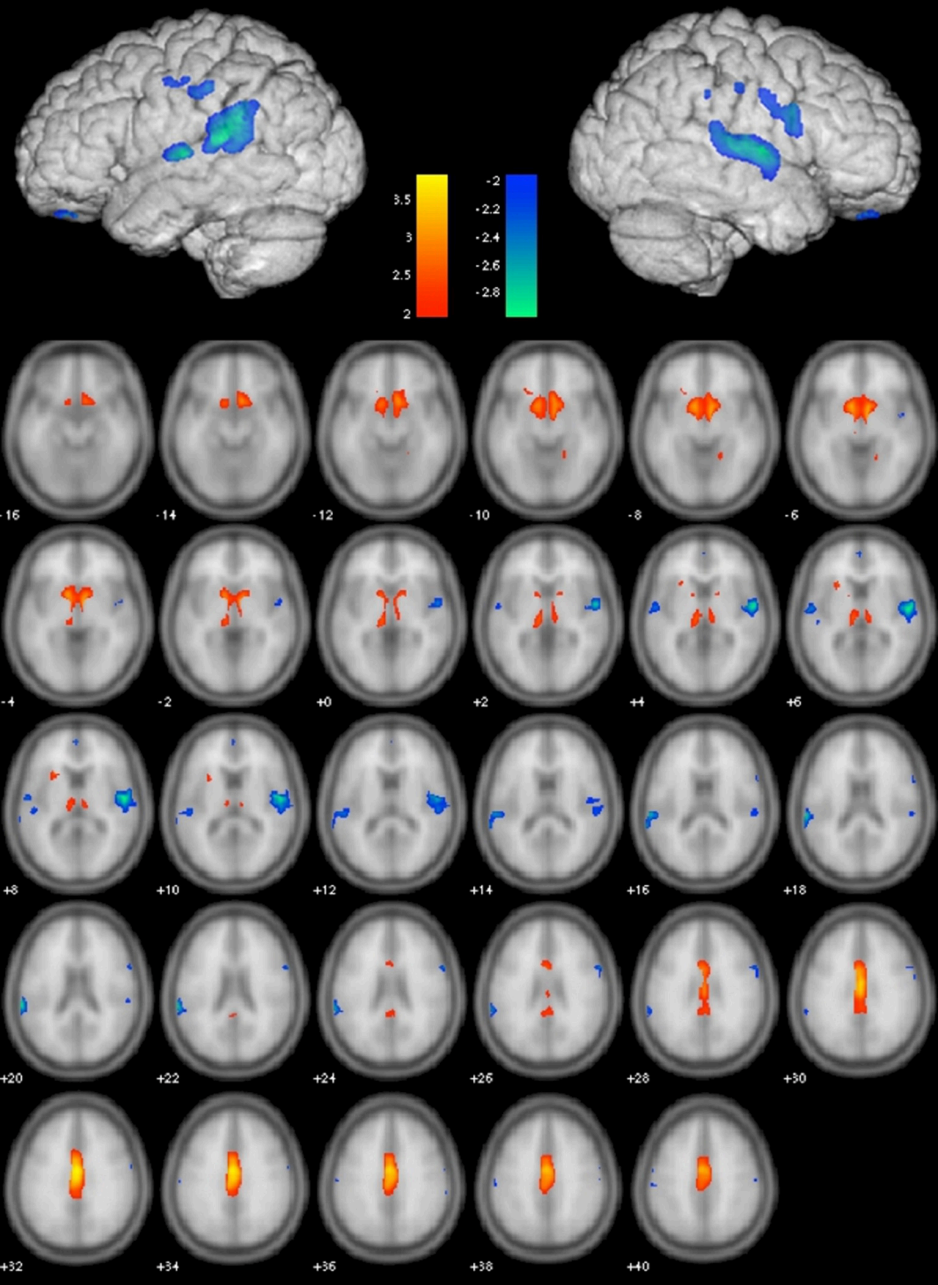
**Gray Matter Volume (GMV) Covariance Pattern predicting log of white matter hyperintensity (WMH) volume across the entire cohort**. Color bars depict Z-scores. Blue shows areas of reduced GMV *Z* ≤ −2 associated with greater WMH while red shows relative volume preservation with *Z* ≥ 2.

Gray matter VBM in combination with SSM and AIC identified the best linear combination of topographical GMV component patterns associated with WMH volumes in the sample. This model included components 1, 2, and 6 and accounted for 34.5% of the variance in logWMH (*R* = 0.613, adjusted *R*^2^ = 0.345; Figure 1). The WMH~GMV pattern included reduced GMV in the superior temporal gyri bilaterally, the left angular gyrus, and bilateral supramarginal and orbital gyri. GMV was relatively greater in the cingulate cortex bilaterally, the medial thalami and the basal forebrain (Figure 2).

**Figure 3:**
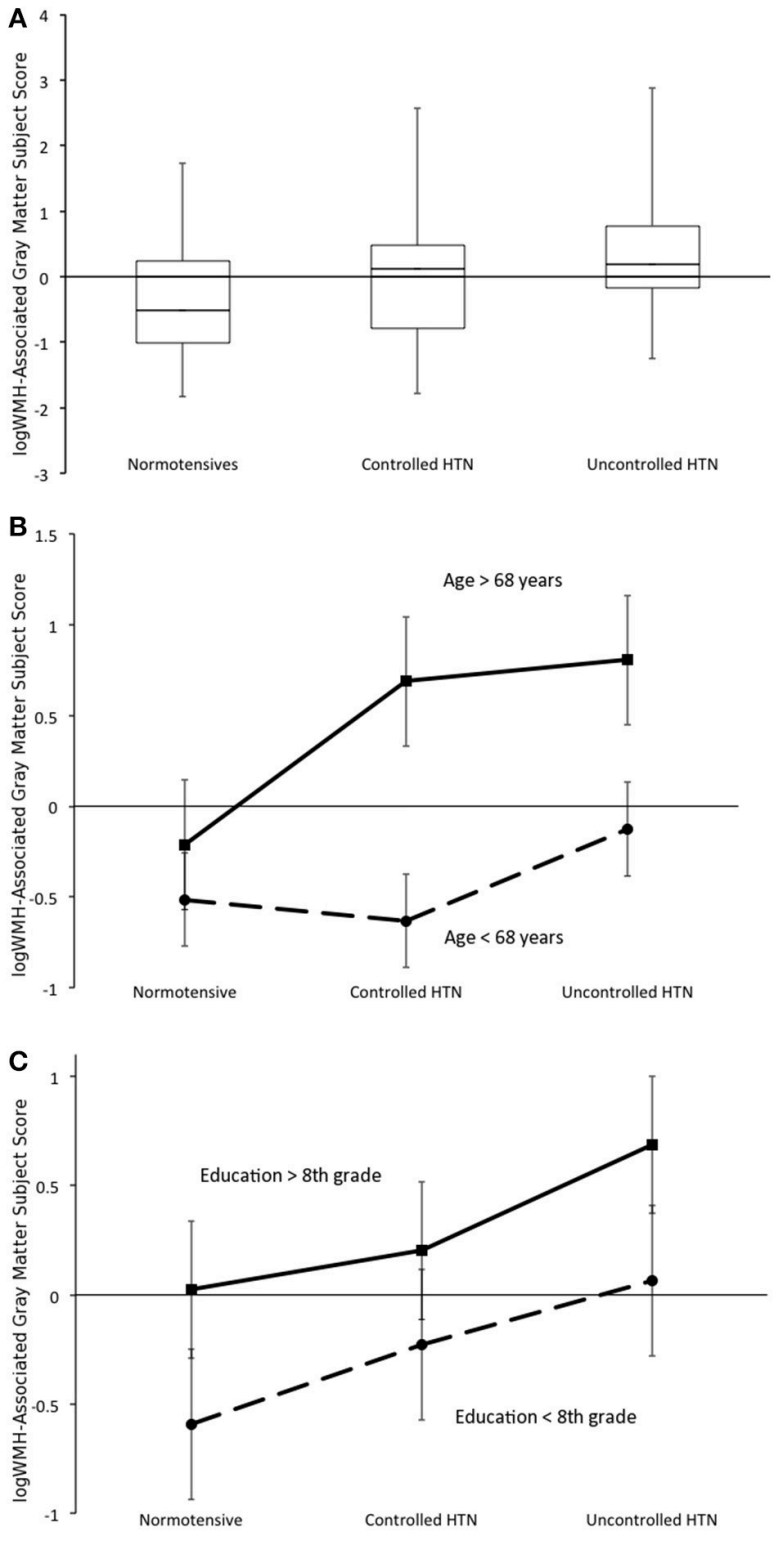
**(A)** Expression of the white matter hyperintensity-associated gray matter volume (WMH~GMV) pattern differs across blood pressure control groups while covarying for age and educational attainment. Boxplot depicts entire range (error bars), 1st quartile, median, and 3rd quartile. **(B)** Main effect of age on blood pressure group x WMH~GMV pattern expression. **(C)** Main effect of education on blood pressure group x WMH~GMV pattern expression interaction. Error bars show 95% confidence intervals **(B,C)**.

In comparing blood pressure control groups, the uncontrolled hypertensive group had the greatest expression of the WMH~GMV pattern (as indicated by the highest pattern subject scores), while the normotensive group had the lowest expression after adjusting for age, gender, education and Gc. The expression of the WMH~GMV pattern was significantly greater in the uncontrolled hypertensive group compared to the normotensive group (ANCOVA: Main effect of group: F-statistic = 4.497, degrees of freedom (dof) = 2, *p* = 0.015), while the difference between uncontrolled hypertensive and controlled hypertensive groups, as well as the difference between controlled hypertensive and normotensive groups were not significant (Figure 3A). Using ordinal regression we tested the linear association between the WMH~GMV pattern and blood pressure group while covarying for age and education and confirmed that blood pressure group predicted expression of the WMH~GMV pattern (*B* = 0.281, adjusted *R*^2^ = 0.064, *p* = 0.024). Neither sex nor Gc showed a significant association with the WMH~GMV pattern.

The WMH~GMV pattern was expressed more in older participants (main effect F-statistic = 26.627, dof = 1, *p* < 0.001), but there was no age by blood pressure group interaction (Figure 3B). Participants with higher education also had greater expression of the WMH~GMV pattern (main effect F-statistic = 7.138, dof = 1, *p* = 0.01), but there was no education by group interaction (Figure 3C).

Using multivariate linear regression, both increasing age (*B* = 0.504, adjusted *R*^2^ = 0.242, *p* < 0.001) and greater educational attainment (*B* = 0.289, adjusted *R*^2^ = 0.069, *p* = 0.021) were associated with greater expression of the WMH~GMV pattern despite having preserved cognitive performance.

**Figure 4:**
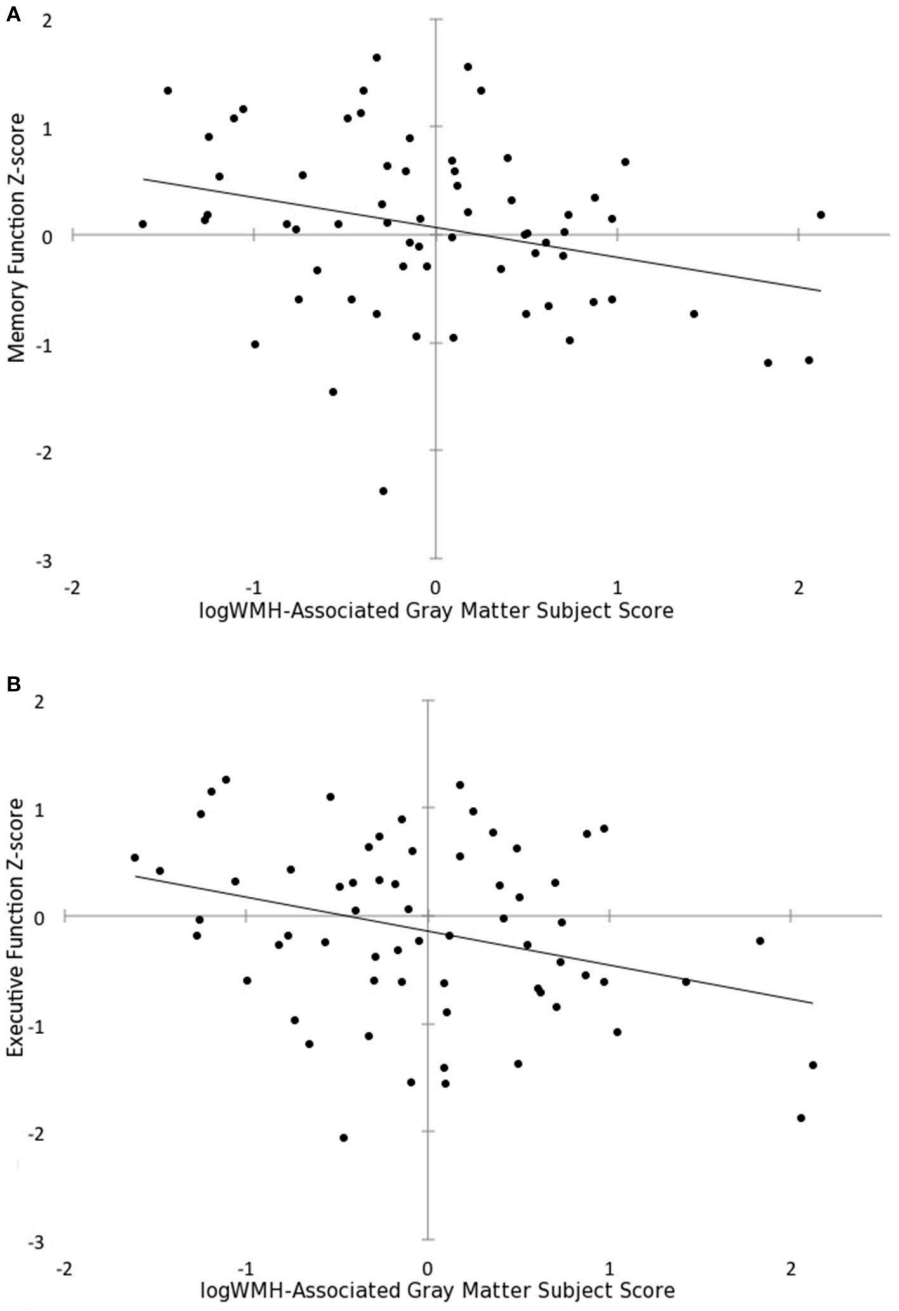
**Subject scores for the white matter hyperintensity-associated gray matter volume pattern predicts (A)** memory function Z-score (*B* = −0.277, *p* = 0.012) and **(B)** executive function Z-score (*B* = −0.315, *p* = 0.004) after adjusting for age and educational attainment.

Greater expression of the WMH~GMV pattern was also associated with worse memory (*B* = −0.277, *p* = 0.012, corrected *p* = 0.048) and executive function (*B* = −0.315, *p* = 0.004, corrected *p* = 0.016) while adjusting for age and education (Figure 4). However, WMH~GMV pattern expression was not associated with processing speed, or language scores. Lower GMF was also independently associated with worse memory (*R* = 0.42, *p* = 0.01), but not executive function, language or processing speed. Cognitive scores were not associated with logWMH or adjusted TBV.

In the subgroup of 58 participants with CESD scores (20 normotensive, 19 controlled hypertensive, 19 uncontrolled hypertensive), depressive symptoms were not associated with WMH, TBV, expression of WMH~GMV pattern, or any of the cognitive domains.

APOE genetic testing was available on 58 of the 64 participants. Twenty-eight percent have at least 1 APOE-4 allele. There were more APOE-4 alleles in the normotensive group (normotensive 7/17, controlled hypertensives 6/21, uncontrolled hypertensives 3/20). Using logistic regression, APOE-4 status was not significantly associated with brain volume, WMH volume, expression of the WMH~GMV pattern, or any of the cognitive domains.

## Discussion

Using a multivariate neuroimaging approach incorporating both gray and white matter imaging techniques, we identified a pattern of GMV differences associated with WMH lesion load in a group of older adults without significant cognitive impairment. Areas of relatively smaller cortical GMV in the bilateral superior temporal and temporal-parietal junctions as well as the orbital cortex may reflect brain regions particularly sensitive to the effects of small-vessel disease. Our finding that this WMH~GMV relationship was strongest in patients with uncontrolled hypertension, and weakest in normotensives supports this conclusion. Previous studies found that uncontrolled blood pressure predisposes to white matter disease, (van Swieten et al., 1991; de Leeuw et al., 2002) and that successful treatment reduces the risk of developing white matter lesions. (Liao et al., 1996) In our study, group differences in WMH volume or TBV were not significant nor predictive, but we identified a regional pattern of GMV differences associated with WMH that provides a potential mechanism for the link between poor blood pressure control, gray matter atrophy and age-related cognitive changes. (Nagai et al., 2008) Other studies have also demonstrated associations between WMH and GM atrophy (Appelman et al., 2009). Raji et al. (2012) found an association between WMH and GMV in a similar pattern involving the frontal and temporal-parietal regions independent of age. We find that poor blood pressure control augments the effect of WMH on GM atrophy, even without differences in WMH volume or TBV.

We also identified areas of relative brain volume preservation across the cingulate gyrus, the basal forebrain and the thalamus that reflect regions less affected by cerebral small-vessel disease, or perhaps indicate areas more strongly influenced by non-vascular mechanisms such as amyloid deposition. In a VBM study of mild cognitive impairment and Alzheimer's disease patients without cardiovascular disease or extensive cerebrovascular disease, Karas et al. demonstrated both thalamic and cingulate cortex atrophy compared to healthy controls (Karas et al., 2004). However most studies identify extensive overlap between risk factors for Alzheimer's disease and WMH. Some theorize that amyloid deposition and vascular damage may act synergistically in the clinical manifestation of dementia (Erten-Lyons et al., 2013; Provenzano et al., 2013).

The effect of age on the WMH~GMV relationship reveals that older individuals may be more susceptible to brain atrophy resulting from small-vessel disease. Indeed previous studies found differential effects of both hypertension and age (Appelman et al., 2009) as well as interactions between them (Strassburger et al., 1997; Raji et al., 2012). Arteriolar sclerosis that occurs with both high blood pressure and aging of the vascular system likely underlies endothelial dysfunction, reduced vasoreactivity, and subsequent ischemia. Chronic ischemia results in both demyelination and possibly Wallerian degeneration (Appelman et al., 2009). The deleterious effects of these vascular changes on GM is less clear, in part because imaging modalities sensitive to WMH are not sensitive to GMV changes. While our findings are cross-sectional, these data raise the possibility that better blood pressure control could prevent cortical atrophy, especially in older individuals, and prospective studies are needed to directly test this hypothesis. The ongoing SPRINT-MIND trial (Systolic Pressure Intervention Trial) may help in this regard (ClinicalTrials.gov: NCT01206062). Meanwhile, evidence from the Systolic Hypertension in Europe (Syst-Eur) trial demonstrated that antihypertensive treatment reduced the incidence of dementia (Forette et al., 1998).

The expression of a WMH~GMV pattern is clinically relevant in our study since it was associated with cognitive performance, while neither WMH volume nor adjusted TBV showed such a relationship. Greater expression of the WMH~GMV pattern, after adjusting for age and education, predicted lower memory and executive function scores, despite our sample being cognitively intact overall. This finding is consistent with previous studies showing an association between memory function and both white (de Groot et al., 2000) and gray matter insult (DeCarli et al., 1995). Raji et al. (2012) concluded that while hypertension was associated with WMH, the cognitive effects of hypertension may be mediated by gray matter atrophy. The association in our study with executive function was even stronger after adjusting for age and education, which is also consistent with previous studies (DeCarli et al., 1995; Prins et al., 2005). Executive dysfunction has previously been associated with age, frontal lobe atrophy and frontal lobe WMH, and has been implicated as a marker for vascular cognitive impairment (Salthouse et al., 2003; Wright et al., 2008). By using a multivariate analysis of structural T1 and T2 differences, we identified a potential neuroimaging biomarker that may be more sensitive to early changes in domain-specific cognition in the elderly than conventional measures such as WMH or TBV alone. Longitudinal studies could evaluate this pattern as a biomarker for cognitive decline in aging. In contrast to previous studies (Ylikoski et al., 1993; Prins et al., 2005) we did not find WMH to be predictive of processing speed, but we were limited to only one processing speed test with low inter-subject variance.

In this community-based sample of older participants with preserved cognition, we found that those with more education had a trend for greater WMH load and significantly greater expression of WMH-associated cerebral atrophy, despite similar cognitive performance. These findings support the idea that education contributes to cognitive reserve, wherein cognitive function is preserved despite structural deterioration (Stern, 2002; Bennett et al., 2003). Cognitive reserve relates to lifestyle characteristics including higher education and occupational complexity (Stern et al., 1995; Bennett et al., 2003; Le Carret et al., 2003), and is thought to reflect greater efficiency in cognitive processes, permitting greater resistance to structural insult. Reserve may also reflect adaptive neural processes and recruitment of additional or more extensive brain areas to circumvent structural pathology (Stern, 2002). Alternatively, genetic predisposition responsible for cognitive resilience may instead permit higher educational attainment.

There are several limitations to this study. We used WMH volume, a summary measure for global white matter damage reflective of small-vessel disease. While easily acquired on commonly available clinical MRI scans, FLAIR WMH are associated with heterogeneous underlying histopathology: from mild perivascular tissue damage surrounding lipohyalinotic arterioles with minimal axonal loss, to more severe ischemic damage with extensive myelin and axonal loss (Gouw et al., 2008). We emphasized hypertensive vascular risk in this study, but WMH may also result from other factors including Alzheimer's disease, alcoholism or head trauma. Furthermore, regional WMH distribution may affect the sensitivity of detection on MRI, as well as alter the extent and pattern of cognitive impairment. Future studies using diffusion tensor imaging may elucidate the link between regional WM insult, gray matter atrophy, cerebrovascular risk factors, and cognition. Finally, we highlight the importance of blood pressure control on preventing brain structural changes associated with cognitive decline. However, our cross-sectional study cannot fully evaluate the effect of the duration of blood pressure control. Nor is it powered to assess the class effects of different antihypertensive medications. Larger longitudinal studies are needed to evaluate optimization of blood pressure treatment to limit structural and cognitive changes.

## Conclusions

We evaluated a race-ethnically diverse, community-based population of older adults without cognitive impairment. In doing so we avoided the selection bias of hospital or referral based studies but controlled many of the known risk factors for WMH, gray matter atrophy, and cognitive decline by matching groups for age, sex, and education to focus primarily on the effects of blood pressure control on these outcomes. Rather than assess WM or gray matter pathology alone, we used an integrative, multivariate approach that evaluated modifiers of a structural relationship between GMV and WMH, as a marker of cerebral small-vessel disease. We identified a pattern of WMH~GMV differences that was sensitive to the effects of blood pressure control and predictive of cognitive performance. We conclude that controlling blood pressure may limit the effects of small-vessel disease and aging on cerebral gray matter atrophy, potentially delaying or preventing the onset of cognitive sequelae.

## Author contributions

KK contributed to data analyses, interpretation and manuscript preparation. CW contributed to study oversight, recruitment, data preparation, analyses, interpretation and manuscript preparation. KB, MF, KC, and NN contributed to data preparation, analyses and interpretation. JM, ME, RS, YS, and CD contributed to study oversight, recruitment, and data analyses and interpretation. GA contributed to study oversight, data analyses, interpretation, and manuscript preparation.

## Funding

CW receives federal grant support (K02 NS 059729, R01 HL 108623, SPRINT MRI WFUHS 330214, R01 NS 29993) and private foundation support (American Heart Association Bugher Center project 14BFSC17690003). He receives royalties from UpToDate for 2 vascular dementia chapters. ME receives compensation for providing consultative services for BioTelemetry/Cardionet, BMS-Pfizer Partnership, Boehringer- Ingelheim, Daiichi-Sankyo, Janssen Pharmaceuticals, and Sanofi-Regeneron Partnership; receives research support from diaDexus Inc., Bristol- Myers Squibb/Sanofi Pharmaceuticals Partnership, and the NIH/NINDS; has given expert legal opinions on behalf of Organon (NuvaRing and stroke litigation) and Hi-Tech; and serves on the National, Founders Affiliate, and New York City chapter boards of the American Heart Association/American Stroke Association. He receives royalties from UpToDate for chapters related to stroke. RS receives federal grant support (R01 NS 29993), private foundation support (American Heart Association Bugher Center), and pharma research support (Boehringer Ingelheim). GA receives federal grant support from the National Institute on Aging (R01 AG049464, P30 AG019610) and funding from the State of Arizona and ADHS, the Advanced Research Institute for Biomedical Imaging, and the McKnight Brain Research Foundation. CD receives federal grant support. YS receives federal grant support. The remaining authors declare no conflicts of interest. The funders had no role in study design, data collection and analysis, decision to publish, or preparation of the manuscript.

### Conflict of interest statement

The authors declare that the research was conducted in the absence of any commercial or financial relationships that could be construed as a potential conflict of interest.
