# Supplementary material for: Blood Pressure Control in Aging Predicts Cerebral Atrophy Related to Small-Vessel White Matter Lesions
Source: Front Aging Neurosci. 2017 May 15;9:132. doi: 10.3389/fnagi.2017.00132 (PMC5430031; doi:10.3389/fnagi.2017.00132)
Supplement: Supplementary file 1 [file Table1.docx]

**Supplemental Table: Antihypertensive Treatment**

| **Controlled Hypertensives (n=22)** | **Uncontrolled Hypertensives (n=21)** |
| --- | --- |
| HCTZ | no antihypertensive |
| HCTZ | no antihypertensive |
| Triamterene | no antihypertensive |
| Amlodipine | no antihypertensive |
| Amlodipine | no antihypertensive |
| Diltiazem | no antihypertensive |
| Verapamil | no antihypertensive |
| Atenolol | no antihypertensive |
| Metoprolol | no antihypertensive |
| Metoprolol | HCTZ |
| Metoprolol | HCTZ |
| Enalapril | Amlodipine |
| Fosinopril | Nifedipine |
| Lisinopril | Nifedipine |
| Lisinopril | Benazepril |
| Losartan | HCTZ, Triamterene |
| Valsartan | HCTZ, Nifedipine |
| Triamterene, Verapamil | Triamterene, Amlodipine |
| HCTZ, Benazapril | HCTZ, Metoprolol |
| HCTZ, Enalapril | Triamterene, Quinapril |
| Diltiazem, Atenolol | HCTZ, Bisoprolol, Losartan |
| Amlodipine, Lisinopril |  |
|  |  |
| *Hydrochlorothiazide = HCTZ* |  |
